# The QALY is ableist: on the unethical implications of health states worse than dead

**DOI:** 10.1007/s11136-021-03052-4

**Published:** 2021-12-09

**Authors:** Paul Schneider

**Affiliations:** School of Health and Related Research (ScHARR), University of Sheffield, Sheffield, 30 Regent St, Sheffield, S1 4DA UK

**Keywords:** Bioethics, Health economics, Health valuation, QALY, States worse than dead, Utilities

## Abstract

**Introduction:**

A long-standing criticism of the QALY has been that it would discriminate against people in poor health: extending the lives of individuals with underlying health conditions gains fewer QALYs than extending the lives of ‘more healthy’ individuals. Proponents of the QALY counter that this only reflects the general public’s preferences and constitutes an efficient allocation of resources. A pivotal issue that has thus far been overlooked is that there can also be negative QALYs.

**Methods and results:**

Negative QALYs are assigned to the times spent in any health state that is considered to be worse than dead. In a health economic evaluation, extending the lives of people who live in such states reduces the overall population health; it counts as a loss. The problem with this assessment is that the QALY is not based on the perspectives of individual patients—who usually consider their lives to be well worth living—but it reflects the preferences of the general public. While it may be generally legitimate to use those preferences to inform decisions about the allocation of health care resources, when it comes to states worse than dead, the implications are deeply problematic. In this paper, I discuss the (un)ethical aspects of states worse than dead and demonstrate how their use in economic evaluation leads to a systematic underestimation of the value of life-extending treatments.

**Conclusion:**

States worse than dead should thus no longer be used, and a non-negative value should be placed on all human lives.

## Introduction

The concept of Quality-Adjusted Life Years (QALYs) is being widely used to inform societal decisions about the allocation of health care resources [1]. By some, it is even considered the ‘gold standard’ for measuring and valuing health in economic evaluations [2]. However, it is not without limitations: a long-standing line of critique has been that the QALY discriminates against people with disabilities and those in poor health [3]: all else being equal, extending the lives of individuals with disabilities or underlying health conditions gains fewer QALYs than extending the lives of ‘more healthy’ individuals. Several authors have argued that this is unjust, and that all life years should be of equal value [4–6].

Proponents of the QALY framework counter that since most people state that they are willing to give up some of their remaining lifetime for improvements in their health-related quality of life (HRQoL), it is only rational that one additional life year in poor health is of lower value than one additional life year in perfect health. Discrimination based on individuals’ HRQoL is then necessary in order to allocate resources most efficiently [7–10].

One pivotal issue that has thus far not been considered in this debate is that HRQoL can not only be low, but also negative: ‘health states worse than dead’ (SWD) get assigned negative values. Extending the live of a person who lives in a SWD generates negative QALYs.

While largely overlooked by previous research, the implications of SWDs are significant. Their use in health economic evaluations implies value judgements that, at closer inspection, appear to be ableist and unethical. Furthermore, they lead to the systematic underestimation of the value of life-extending treatments in almost any patient group. In this paper, I thus argue that the concept of SWD should be abandoned.

The sophistication and complexity of health economic evaluations can make it difficult to examine their implicit value judgements [11]. The remainder of this paper thus begins with a background section, in which some key concepts are revisited (“Background” section). In “Motivating example—Step I” section, a very simple motivating example is provided, which is used to develop some intuition for the ethical implications of SWD. The subsequent section (“SWD and the conflict between individual and social preferences” section) is a brief digression to clarify potential misconceptions about social value sets. Only then I expand the example from “Motivating example—Step I” section, to demonstrate the, perhaps somewhat intuitive, effects of SWD on the group-level (“Motivating example—continued” section). In “Discussion and further considerations” section, I discuss the implications of and solutions for the issues raised.

## Background

### The valuation of health

The QALY is defined as the arithmetic product of survival time and HRQoL. HRQoL, in turn, is determined by the health state an individual is living in. This means, ‘measuring’ QALYs usually involves two components: firstly, a set of health states; and secondly, numeric scores that reflect their respective desirability. These values are often also referred to as utilities, social values, preference-, (health-related) quality of life-, or QALY-weights. Customarily they are supposed to reflect the preferences of the general public [12].

There are many different ways to classify health states (such as EQ-5D, SF-6D, or HUI), and various methods to derive numeric score/social values for them (such as time trade-off (TTO), standard gamble, or discrete choice experiments) [13]. The arguments of this paper are relevant to all of them, but for simplicity, I will only refer to EQ-5D-3L system and the TTO method, as those are currently used as the reference case in the UK [14].

In a TTO exercise, individual preferences for health states are elicited by identifying points of indifference between a longer life in poor health, and a shorter life in perfect health [15, 16]. Preferences are measured in terms of utility values on a scale that is anchored at perfect health, which is assigned a value of 1, and dead, which is assigned a value of 0. The social value of any health state is then constituted by the average utility [17, 18].

### Negative utilities for SWD

If an individual states that they prefer immediate death over living any amount of time in state *j*, this state is considered to be worse than dead. The point of indifference is then derived from the number of life years in full health a person would be willing to give up to avoid living in that state for a certain number of years. If, for example, a person is indifferent between living 5 years in perfect health (followed by death), and living 10 years in perfect health, followed by 10 years in some health state *j* (then followed by death), it is inferred that state *j* has a utility of − 0.5 ($5\times 1 \sim 10 \times 1 + 10 \times j = > j = -0.5$).

It may be interesting to note that negative utilities have different characteristics than their positive counterparts. Positive utilities are measured as a proportion of the utility for full health, with an upper limit of 1. Negative utilities are much harder to interpret and have no limit. Theoretically, they can take the value of minus infinity. In practice, this can cause problems, because very low negative values can have significant impact on the estimation of the average utility values. To limit their influence, negative utilities are usually constrained (rather arbitrarily) to a lower limit of − 1, either by choosing an experimental design that does not allow for lower values, or by rescaling lower negative values, after they are collected [19, 20].

## Motivating example—Step I

Suppose Alice has a severe health condition called *D*, and, according to some social value set, her health state has a value of − 0.1. With the current standard treatment (alternative A), she will be able to live 10 years in her current state before she dies. Now, suppose a new treatment (alternative B) becomes available, which prolongs Alice’s life by 10 more years, i.e. giving her 20 years in total, but it has no effect on HRQoL. Further suppose that the new treatment costs exactly the same as the old treatment—it does not incur any additional costs.

An economic evaluation that weighs the costs and the benefits of the two alternatives will come to the conclusion that, compared to the old treatment A, the new treatment B generates − 1 QALY at no cost (see below). This means, alternative B is not only not cost-effective, but it is dominated by A. Assuming a threshold of $\pounds$ 20,000 per QALY, the new treatment would need to save more than $\pounds$20,000, before it would be considered cost-effective [21]. Based on this economic evaluation, the recommendation would unmistakably be not to provide the new treatment to Alice.

$$\begin{aligned}&{\Delta }Q_\mathrm{B}=\frac{c_\mathrm{B}-c_\mathrm{A}}{s_\mathrm{B}*q_\mathrm{B}-s_\mathrm{A}*q_\mathrm{A}}=\frac{0}{(-0.1)*20-(-0.1)*10}\\&\quad =\frac{0}{-1}\rightarrow \mathrm {dominated} \end{aligned}$$

$\Delta Q$ is the incremental cost-effectiveness ratio; *c* is the costs; *q* is the HRQoL; *s* is the survival time; subscripts A and B indicate the respective alternatives.

The outcome of the economic appraisal seems striking. The new treatment would extend Alice’s survival time by 10 years, it is available at no extra cost, and Alice might be desperate to receive the treatment, yet, society considers Alice’s health state to be worse than dead. Based on this evaluation, the treatment is withhold from her.

It seems obvious that, in this simple example, the value judgement implicit in SWD is unethical. The negative HRQoL suggests that Alice’s health state is worse than dead—but maybe not for her. As a matter of fact, Alice herself might well enjoy life [22]. Even if her health state causes severe suffering, there might be numerous other good reasons for her to seek life-extending treatment (faith, meaning, family, etc.). It should be self-evident that it is not for society to decide whether or not Alice’s life is worth living. To do so would be a blatant violation of her autonomy [23–25]. If she is willing to receive the life-saving treatment, society seems to have no right to deny its provision.

Note that this only holds unequivocally if the new treatment is not more expensive than the old treatment. If the treatment were more costly, the question if, and if so, how much society should be willing to spend to save Alice is a separate issue. It might then be legitimate to decide that saving Alice is not the most efficient use of resources. Yet, given that society is willing to pay for the current treatment, it would be unethical to withhold the new treatment from her.

## SWD and the conflict between individual and social preferences

Before we further expand the example, it will be useful to clarify some potential misconceptions about the type and the admissible domain of the preferences that underlie social value sets/HRQoL values and the QALY.

Generally, social value sets are based on the preferences of the general public [26]. In fact, most national HTA agencies make this explicitly the reference case for health economic evaluations—one notable exception is Sweden, which uses patient preferences (see below) [27]. In a publicly financed health care system, this seems desirable from a democratic perspective. Citizens—sometimes confused with ‘taxpayers’ (e.g. [28])—should have some say in decisions about the allocation of health care resources [18, 26]. It may thus be legitimate to use health states preferences of the general public to inform societal decision-making. When it comes to SWD, however, the preferences of the general public are (1) ill-informed, (2) misconstrued, and/or (3) irrelevant. In the following, I shall further elaborate on these three points.

1. Ill-informed: The preferences of the general public do not correspond to patients’ evaluation of their own situations; they should not be confused with a measure of patients’ self-assessed HRQoL.

Members of the general public usually have little or no experience with severe health problems. When asked to imagine living 10 years with impaired mobility, for example, they tend to focus on the immediate negative impact that the loss of mobility might have of their life now. Yet, they fail to consider all the other relevant aspects that do not change—or even improve. As a result members of the general public generally overestimate the impact of health impairments. They give significantly lower health state utilities than people who actually live in those health states [29].

The Swedish, experience-based value set demonstrates the difference very clearly. For this study, Burström et al. [30] asked about 45,000 individuals in Sweden to value the (EQ-5D-3L) health state they are currently in, using the TTO method. The experience-based value set they derived is strikingly different from value sets that are based on the preferences of the general public, in that it did not contain any SWD. With a value of 0.34, even the worst health state had a relatively high value.

For comparison, the UK social value set (which is based on the preferences of the general public) contains 84 SWD—that is 34.6% of all the 243 health states that the EQ-5D-3L system can describe [31, 32]. The proportion of SWD varies greatly between countries, ranging from 2% in Zimbabwe to 60% in Singapore [33, 34]. According to the UK social value set, about 1.5% of the adult population in England, that is approximately 840,000 individuals, are currently considered to be living in a SWD (own analysis, [35]). Among patients, the proportion is likely to be much higher.

On a side note, it should be mentioned that people’s adaptation to poor health and disability are sometimes also viewed as problematic. It is argued that patients’ utility values could be higher only because of lowered expectations, cognitive denial, or some other bias, that leads patients to underestimate how much they would benefit from improvements of their health states. It may then not be desirable to take patients’ utilities at face value [36]. Nonetheless, in the context of SWD, this argument seems hardly plausible. If a patient thinks their life is worth living, it would be absurd to consider them factually mistaken, and to maintain that they are objectively better off if they were dead.

2. Misconstrued: Social value sets do not reflect the general public’s preferences for the allocation of resources.

It could be argued that social value sets are not supposed to reflect how individuals experience certain health states, but to reflect social preferences for the allocation of health care resources [29]. If that is the case, social value sets are falsely constructed and clearly misspecified.

Participants in health valuation studies are not asked how they prefer resources to be allocated. This would require using a method like the person trade-off, for example, in which participants are asked to make choices about two groups of people, which differ in size and in their health states [37]. Instead, TTO or SG are used, which ask participants to imagine being in a particular health state themselves. Yet, this one type of preferences can not easily be translated into another. Some people may, for example, say that they would rather prefer to be dead, than to be confined to bed [38]. Yet, the very same people will probably consider their preferences being misrepresented, if they led to the evaluation that people who are confined to bed should not be offered life-extending treatments. They may rightly object that this is just not what they meant.

3. Irrelevant: Even if social value sets would accurately reflect the general public’s preferences, in the context of SWD, those should be considered irrelevant.

It seems rather improbable that members of the general public in the UK, or anywhere else for that matter, would actually support the concept of SWD and their implicit value judgement—which we will discuss in more detail in the next section. However, even if some individuals wanted some other individuals to die earlier rather than later, those preferences should be deemed irrelevant for treatment reimbursement decisions.

While everyone has, of course, the right to consider their own life in a certain health state to be worse than dead and to refuse life-extending treatments, considering someone else’s life in a certain health state worse than dead is morally a completely different issue. To then also prefer that life-extending treatments are withheld from certain (other) individuals, because one prefers them to be dead, would undoubtedly be reprehensible. It would constitute an objectionable preference [11, 23].

To clarify, this paper neither tries to argue that SWD do not exist, nor to promote treating people in poor health states, who do not want to be treated. The focus of this paper is on societal reimbursement decisions—i.e. should a given life-extending treatment be made available in the health care system, in case an individual seeks it. Whether or not their life is worth extending, and the treatment is actually taken, is for to the individual to decide. If they do not wish to prolong their lives in poor health states, they can, of course, refuse to take the treatment and/or choose to stop the treatment at any time [25]. The point I am trying to make is that whether the general population considers these health states better or worse than dead should be considered irrelevant in this context.

Of course, in some cases individuals are not able to express their own will (e.g. young children, unconscious patients, etc.), which often poses complex ethical challenges. Yet, these lie outside the scope of this paper and will not be discussed here. It should only be noted that in these situations, decisions ought to be made on the individual’s behalf (‘what would they have decided?’)—Social health state values, which are based on the preferences of the general public, do not appear to be particularly helpful to inform such decisions.

In liberal societies, individual rights set boundaries for the realisation of preferences and constrain what can be done in pursuit of collective interests. This means, restrictions are imposed on the domain of preferences to protect individual rights. Certain types of preferences, say for sexism, racism, genocide, or tyranny, are being discarded as objectionable and ignored in societal decision-making: it just does not matter how many people prefer that health care is only provided to people of a certain ethnicity or how strong their preferences are. Such views are simply not taken into account. This means, even if some individuals preferred that some other individuals in SWD do not get access to life-extending treatments, their preferences should be considered objectionable and be discarded.

## Motivating example—continued

### Step II

The example given above may not seem particularly relevant, as QALYs are not evaluated on the individual-level. Treatment reimbursement decisions are, accordingly, also not made for single individuals, but only for groups. However, by incrementally expanding the simple example I will try to show that the intuition developed for the individual case also applies to the aggregate level. That is to say, if one accepts that it would be unethical to withhold the life-extending treatment from Alice in the example above, it follows that one also has to reject the use of SWD in health economic evaluations altogether.

To value health outcomes for a group, HRQoL values are aggregated, across many different individuals and over time. The resulting ‘disease state utilities’ usually reflect the average HRQoL of a group of patients with some disease. Commonly used disease states include, for example, ‘pre-progression’ and ‘post-progression’ in lung cancer; or ‘mild’, ‘moderate’, and ‘severe’ in COPD.

If now there was a group of individuals, of which all, like Alice, live in SWD, it seems obvious that the arguments made above still apply. That is to say, if one accepts that society should provide life-extending treatment for Alice—if the treatment is not more expensive than the current standard of care—society should obviously do the same for each member of the group.

Now we will take the scenario one step further and show that SWD can also have significant implications for individuals who live in states that are better than dead (SBD), and that they can affect decisions for new treatments that are more costly than the current treatment.

### Step III

Suppose Bob, and Claire are a group of patients with some chronic disease *D*. They live in health states with HRQoL values of $+\,0.2$, and $+\,0.4$, respectively. The average HRQoL for disease *D* is then given by $\frac{0.2 +0.4}{2} = 0.3.$ With the current standard treatment, they are both expected to live 10 years before they die.

Further suppose that a new life-extending treatment *C* becomes available (again, with no effect on HRQoL), which prolongs the lives of patients with disease *D* by another 10 years, i.e. giving them 20 years in total. The treatment is if $\pounds\, 19,000$ more expensive than the standard treatment.

Still assuming a threshold of $\pounds\, 20,000$ per QALY, we can derive an incremental cost-effectiveness ratio (ICER) of $\frac{\pounds\, 38,000}{0.3 \times 10}= \pounds 12,667$ per additional QALY. Consequently, treatment *C* would be considered cost-effective.

### Step IV

Suppose that Alice, still living in a state with a HRQoL of $-\,0.1,$ also has disease *D*, and that she joins the group of Bob and Claire. The average HRQoL for disease *D* is then given by $\frac{-0.1 + 0.2 +0.4}{3} = 0.167.$ Now, the ICER increases to $\frac{\pounds\, 38,000}{0.167 \times 10}= \pounds \,22,754$ per QALY and treatment *C* suddenly is no longer cost-effective.

This evaluation should be considered unethical. The average utility value of 0.167 reflects a mixture of Bob’s and Claire’s positive, and Alice’s negative HRQoL values. Thereby, the willingness to pay for an additional life year in that group is reduced proportional to Alice’s negative HRQoL. The implications are significant: Treatment *C* is not provided to the patients with disease *D*, only because society prefers Alice to die sooner rather than later—the decision is made *as if* Alice’s life were considered unworthy of living.

If society were indifferent whether Alice dies or lives, i.e. her health state had a value of 0, the treatment would become cost-effective. The average HRQoL of disease *D* would then increase to $\frac{0 + 0.2 +0.4}{3} = 0.2,$ and the ICER would drop under 20,000 again, with $\frac{\pounds\, 38,000}{0.2 \times 10}= \pounds\, 19,000$ per QALY. What this result suggests is health economic evaluations may systematically underestimate the value of any life-extending medical intervention.

## Discussion and further considerations

This paper has demonstrated that when SWD are used to value changes in survival times, they imply unethical value judgements and discriminate against those people in poor health states. This holds true, regardless of whether SWD occur on the individual-level, where they are immediately visible, or on the group-level, where they may be hidden within an aggregate average. I thus argue that SWD should not be used in health economic evaluations. Extending a person’s life should generate at least zero QALYs, and shortening should not gain any QALYs, respectively.

This position does not seem to be controversial: while there may be reasonable disagreement over the relative value of life years gained in one group compared to another, an additional life year should never be considered a loss for society in itself. Yet, as the examples in this paper have shown, this is exactly what SWD imply. It therefore seems striking how widely and uncritically SWD have been and are being used in health economic evaluations. It can only be attributed to the complexity of economic modelling, which may conceal the implicit value judgements, that there has not been an outrage from the general public, patient advocacy groups, and/or health economists.

Some may argue that it is not immediately clear if, and if so, to what extent the thesis of this paper applies to decision-making in the real world. HTA agencies surely will recognise that it would be deeply problematic to estimate the QALY gains from, say, providing feeding tubes for children with severe birth defects, or mechanical ventilation for patients with advanced amyotrophic lateral sclerosis. Life-extending treatments like these for people in severe health states are likely to be provided, even if they are clearly not cost-effective (according to the current QALY framework). This means, the arguments raised in this paper are mainly relevant to those cases where the unethical implications of the QALY framework are not obvious; where the QALY losses from extending the lives of people in SWD are concealed from the decision makers. SWD may then lead to an underestimation of the value of a life-extending treatment. People in SWD living for longer cause the average ICER estimate to be higher, without anyone noticing it, and, most importantly, (presumably) without anyone’s intention for it to be the case.

I would like to stress that the ICER estimates of almost any life-extending treatment can potentially be affected by SWD. As mentioned above, SWD are not uncommon: 1.5% of the English adult population lives in a SWD. The prevalence among patients can be assumed to be much higher, but detailed information on SWD is scarce. In some rare instances, SWD can be spotted directly by inspecting the economic model. To give but one example, in NICE’s 2019 appraisal of Nusinersen for treating spinal muscular atrophy, four of seven non-dead states had a negative value in the reference scenario for one of the subgroups—increased survival time in these states led to a lower QALY estimate [39]. However, most often, one will need to assess the disaggregated data on patients’ self-reported health states to identify SWD in the underlying patient population, because even if aggregate utility scores are positive, they may well be affected by SWD: Scott et al. [40], for example, report a median utility score of 0.36 in a sample of 2073 patients awaiting total hip arthroplasty. Yet, they also found that 18.9% of the patients reported to live in state with a negative utility value. Unfortunately, this information is usually not disclosed separately, and so the magnitude of the effect remains largely unknown.

On the other hand, there does not seem to be any compelling reason to use SWD in health economic evaluations to value additional survival time in the first place. SWD neither reflect the preferences of individual patients, nor can they be considered to represent the general public’s preferences for the allocation of health care resources—so why are we using them?

It should be noted that SWD can also give people in poor health states an advantage. Moving someone from a SWD to full health for, say one year, actually generates more QALYs than extending the life of someone living in full health by one year: in the UK EQ-5D-3L social value set, the former is worth 1.59 QALYs; the latter only 1 QALY. This means, for treatments that mainly effect HRQoL, the arguments presented in this paper may indeed not apply. However, the advantage SWD give to some people does not justify the disadvantage they give to others. For treatments that affect both, length and health-related quality of life, it may also be very difficult to determine what the overall effect of SWD is. I thus maintain that, if it cannot be ruled out that some person’s gain in survival time is valued as a loss to society (or vice versa), SWD shall not be used in health economic evaluations.

I would like to emphasise that assigning a non-negative value to all human lifetime should be considered a *minimal* ethical constraint [41]. There are many other, compelling, more fundamental critiques of the QALY metric and its ethical implications. Some have argued, for example, that all human life should have a positive (and just a non-negative) value [42], or that all human life should be of equal value [9] (see below). Admittedly, these proposals are only concerned with methodological details, while the QALY appears to be accepted as a valid point of reference. Yet, the utilitarian QALY framework itself is not value-free, and could also be called into question [4, 7, 43–45]. However, the argument presented in this paper is deliberately presented within a narrowly defined QALY framework. Even if one accepts the QALY framework in general, I would argue that one has to reject the concept of SWD as unethical.

### Moving forward

While I argue for abolishing the use of SWD in health economic evaluations, I do not intend to prescribe a particular approach on how to replace them. Within the QALY framework, there are primarily two options that should be considered.

Firstly, the QALY metric itself could be adjusted, to ensure that every person’s lifetime has some positive, or at least non-negative, value. The Equal Value of Life (‘EVL’) approach, proposed by Nord et al. [9], could be used for this, or the Health Years in Total (‘HYT’) framework, proposed by Basu et al. [42]. The former assigns every additional life year a value of one QALY, while the latter also takes into account HRQoL changes that occur during additional life years. However, both approaches add something extra to the QALY, which is not derived from the social value set, but imposed rather post-hoc by the researcher or decision maker.

The second alternative may thus seem more attractive: preferences could either be elicited from patients/people living in the health states themselves, or a different perspective could be used when eliciting preferences from the general public. The person trade-off method may have some appeal in this context, as it seems to come closest to the type of decision that social value sets actually inform [26, 37]. Both approaches are likely to generate much higher and probably exclusively positive health state values [17, 18, 30].

The question, which approach is most appropriate, cannot be answered in isolation, but must be guided by a normative theory of the valuation of health. Any alternative approach may also come with a number of wider, potentially unintended implications, which need to be considered. In the current absence of a widely accepted, coherent theoretical framework, more conceptual research seems to be needed. In particular, this should include two different strands: firstly, there should be more engagement with fundamental questions about the ethical underpinning of the QALY framework; and, secondly, health economists should enter into a meaningful and sustained dialog with citizens, policy makers, and other stakeholders, to ensure that their methods reflect the norms and values of society. However, it is unlikely that all considerations a society considers to be relevant can ever be operationalised and integrated into a coherent, formal decision analytical framework. It therefore seems essential that the results of any health economic model are checked and qualitatively scrutinised. Health policy decision makers should critically assess the underlying assumptions and their ethical implications. Greater involvement of patients, patient representatives, and carers may help to ensure that their perspectives are accounted for in the decision-making process.
