# Correction to: The QALY is ableist: on the unethical implications of health states worse than dead

**DOI:** 10.1007/s11136-024-03717-w

**Published:** 2024-06-22

**Authors:** Paul Schneider

**Affiliations:** School of Health and Related Research (ScHARR), University of Sheffield, 30 Regent St, Sheffield, S1 4DA UK

**Correction to: Quality of Life Research (2022) 31:1545–1552**

10.1007/s11136-021-03052-4

This correction addresses an error in the second paragraph in the ‘Step III’ section on page 1549. The respective sentence discusses the incremental cost-effectiveness ratio of a hypothetical treatment. It incorrectly states that ‘[t]he treatment is if £ 19, 000 more expensive than the standard treatment’. The subsequent equation, however, uses incremental costs of £38,000, and the remainder of the argument is also based on this figure. I acknowledge that the £19,000 figure was a typographical error and cannot be reconciled with the equation or the subsequent analysis. I apologise for this oversight and any confusion it may have caused. I am grateful to Megan MacKay for spotting this error.

The second sentence of the second paragraph in the ‘Step III’ section on page 1549 should be replaced with the following: ‘The treatment is £38,000 more expensive than the standard treatment’.

